# Emerging *BRAF* Mutations in Cancer Progression and Their Possible Effects on Transcriptional Networks

**DOI:** 10.3390/genes11111342

**Published:** 2020-11-12

**Authors:** Magdalena Śmiech, Paweł Leszczyński, Hidetoshi Kono, Christopher Wardell, Hiroaki Taniguchi

**Affiliations:** 1 Institute of Genetics and Animal Biotechnology, Laboratory for Genome Editing and Transcriptional, Regulation, Polish Academy of Sciences, 05-552 Jastrzębiec, Poland; m.smiech@igbzpan.pl (M.Ś.); p.leszczynski@igbzpan.pl (P.L.); 2 Molecular Modeling and Simulation Group, Institute for Quantum Life Science, National Institutes for Quantum and Radiological Science and Technology, Kizugawa, Kyoto 619-0215, Japan; kono.hidetoshi@qst.go.jp; 3 Department of Biomedical Informatics, University of Arkansas for Medical Sciences, 4301 W Markham St, Little Rock, AR 72205, USA; CPWardell@uams.edu

**Keywords:** BRAF, MAPK/ERK, oncogene, mutations, transcription factors, cancer

## Abstract

Gene mutations can induce cellular alteration and malignant transformation. Development of many types of cancer is associated with mutations in the B-raf proto-oncogene (*BRAF*) gene. The encoded protein is a component of the mitogen-activated protein kinases/extracellular signal-regulated kinases (MAPK/ERK) signaling pathway, transmitting information from the outside to the cell nucleus. The main function of the MAPK/ERK pathway is to regulate cell growth, migration, and proliferation. The most common mutations in the *BRAF* gene encode the V600E mutant (class I), which causes continuous activation and signal transduction, regardless of external stimulus. Consequently, cell proliferation and invasion are enhanced in cancer patients with such mutations. The V600E mutation has been linked to melanoma, colorectal cancer, multiple myeloma, and other types of cancers. Importantly, emerging evidence has recently indicated that new types of mutations (classes II and III) also play a paramount role in the development of cancer. In this minireview, we discuss the influence of various BRAF mutations in cancer, including aberrant transcriptional gene regulation in the affected tissues.

## 1. Introduction

In the human genome, the B-raf proto-oncogene (*BRAF*) gene is located on chromosome 7 (7q34) and encodes the BRAF protein, which is composed of 766 amino acids. While all RAF proteins can phosphorylate MEK (MEK1 and MEK2), BRAF has the strongest activation capacity. Accordingly, the mitogen activated protein kinase/extracellular signal regulated kinase (MAPK/ERK) signaling pathway (also known as the Ras-Raf-MEK-ERK pathway) is stimulated and thus regulates cell proliferation, differentiation, and apoptosis, in response to extracellular stimuli, such as cytokines, growth factors, hormones, and environmental stressors. Activation of Raf-MEK-ERK pathway is also known to stimulate the expression of many target genes [1,2,3]. ERK (ERK1 and ERK2) is activated by MEK in the cytoplasm and transported in the nucleus of cells. In the nucleus, ERK1/2 activates through phosphorylation of many transcription factors [4,5,6]. Dysregulation in the MAPK/ERK cascade due to mutations in constituent proteins of this pathway, including RAS (KRAS and NRAS) and RAF (BRAF), is associated with many types of cancer [7,8].

**Figure 1:**
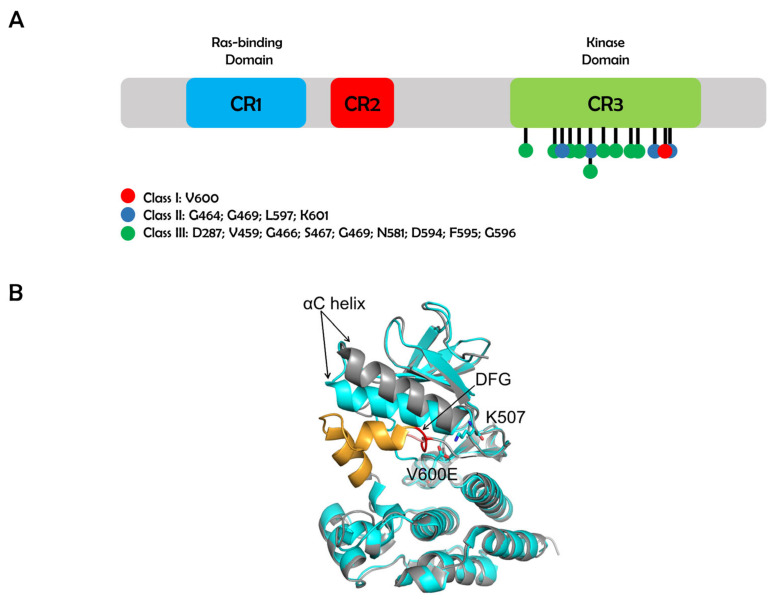
B-raf proto-oncogene (BRAF) protein structure. (**A**) Positions of typical three classes of BRAF mutations are presented. BRAF mutations are indicated in the human BRAF protein structure (CR1; blue, CR2; red, CR3; green). (**B**) Active (cyan, Protein Data Bank (PDB) code 4MNF [16]) and inactive (gray and orange, PDB code 3TV6 [13,16,17]) forms of BRAF. In the active form, E600 (mutation of V600) and K507 form a salt-bridge that drastically changes the position of the αC helix and destabilizes the activation segment (AS, shown in orange). Asp-Phe-Gly (DFG) motif is shown in red.

**Figure 2:**
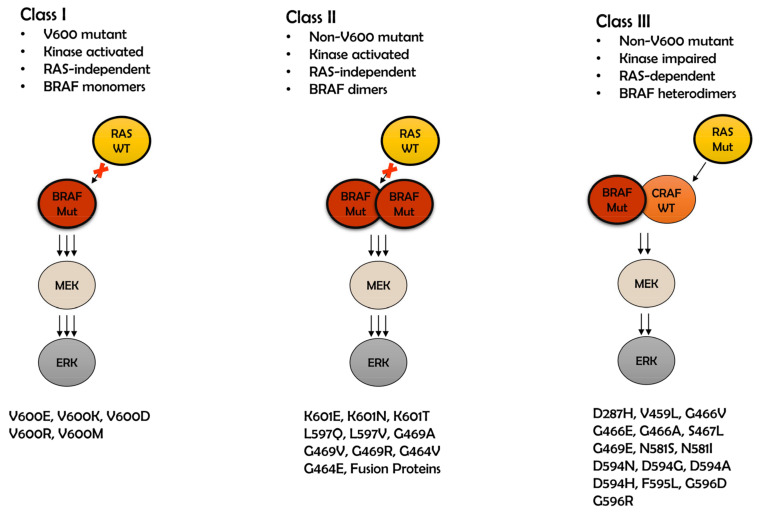
The classification of BRAF mutations and their signaling pathways. Class I is related to codon 600. BRAF V600 (BRAF Mut) acts as a monomer in an RAS-independent manner (RAS WT) and constitutively activate extracellular signal-regulated kinases (ERK) by phosphorylation. In class II signal transduction involves non-V600 mutant. Strong kinase activation is regulated by dimers of mutant BRAF (BRAF Mut), independently of RAS (RAS WT). Class III is kinase-impaired and consists of BRAF non-600 mutant (BRAF Mut) and CRAF wild type (CRAF WT) as a heterodimer. The signal is transferred downstream in the presents of RAS mutant (RAS Mut).

BRAF has three highly conserved domains (CR1, CR2, and CR3). CR1 and CR2 are regulatory regions located toward the ***N***-terminal of the protein. CR1 encompasses the RAS-binding domain (RBD), which interacts with RAS and the cysteine-rich domain (CRD) that binds two zinc ions. CR2 is a serine/threonine rich domain with a 14-3-3 binding site. CR3 has a kinase domain that is located on the C-terminal and is regulated through phosphorylation (Figure 1A) [9,10,11]. The BRAF kinase domain is distinguished by two sections: a small ***N***-terminal lobe and a large C-terminal lobe. The small lobe contains a glycine-rich ATP-phosphate-binding loop, which is also known as the P-loop. In the inactive BRAF kinase state, a crystal structure [12] shows that the P-loop and the activation segment with Asp-Phe-Gly (DFG) motif are positioned near each other and likely stabilized due to an array of hydrophobic interactions, while in another structure [13] the activation segment forms α-helices before the DFG motif (Figure 1B). Phosphorylation of the activating loop leads to BRAF activation. As a result, the hydrophobic interactions or the α-helices are destabilized and the conformational change are induced. This eventually makes the catalytic cleft available. Among all the proteins of RAF family members, BRAF is the main activator of MEK kinases. This is due to BRAF’s constant phosphorylation at residue S446 and negatively charged aspartic acid residues at position D448 and D449. These later residues are in the ***N***-region, which results in a negative charge and facilitates its activation through RAS [7,14]. In contrast to BRAF, CRAF and ARAF require enzyme-catalyzed residue phosphorylation for activation [15]. The BRAF kinase activation segment is evolutionarily conserved among many species. More importantly, mutations of BRAF are found in many forms of cancers and the mutations are classified into three subtypes according to their activation pathways (Figure 2). Although the mechanism of how the mutations induce malignancy is different in terms of their interaction with RAS pathway and partners to form dimers, they all are known to activate ERK phosphorylation. In this review, we will discuss potential transcriptional networks that can mediate BRAF mutation signal to cause malignancy and disrupted gene regulations in the presence of BRAF mutation in several cancers (Table 1 and Table 2).

## 2. Class I BRAF Mutations

There are three classes of BRAF mutations [22] as shown in Figure 2. Class I includes the BRAF V600E mutations and allows the BRAF to act as a constitutively active monomer. Class II mutations allow for constitutively active dimers. Class III either has impaired kinase activity or is inactive [23]. Tumor progression is associated with gene aberrations that modulate cell proliferation, differentiation, survival, and apoptosis. In particular, the accumulation of pathological changes in the proto-oncogenes leads to significant cellular defects [24]. Mutations in BRAF, NRAS, and KRAS components of the MAPK/ERK signaling pathway are frequently identified in melanoma, colorectal, multiple myeloma (MM), papillary thyroid, lung, and ovarian cancers [25,26,27,28,29,30]. BRAF is a major oncogenic driver and therefore a therapeutic target for drug development [31]. Nearly 7% of human cancers are associated with mutations in BRAF, and more than 90% of observed mutations of BRAF are the V600E mutation [32]. Replacement of valine (V) by glutamic acid (E) at position 600 causes a 500-fold increase in kinase activity and leads to increased cell proliferation [12]. This mutation activates BRAF as a monomer, while in the wild type, the dimer formation is required for the activation [16]. It is structurally shown that BRAF with V600E mutation forms a salt-bridge with residue K507 and stabilizes the active form which it is allosterically adopted only upon dimerization of the wild type (Figure 1B) [16,17]. In addition, V600 is part of a hydrophobic cluster that stabilizes the inactive conformation [13,17]. Dysregulation of the MAPK/ERK signaling pathway is caused by upregulated BRAF activity or impaired kinase activity depending on the BRAF mutation locus [8].

## 3. Class II BRAF Mutations

Class II mutations, which are RAS-independent kinase activating dimers, contain K601E, L597Q, and G469A. Notably, it has been demonstrated that 13% of BRAF mutations found from 8405 non-small cell lung cancer patients are G469 mutations [23]. Class II mutations exhibit lower ERK phosphorylation activity than BRAF V600E mutation [12]. Class II mutations are mainly located in the activation segment (K601, L597) or P-loop (G464, G469), and the mutations in this location block self-inhibitory mechanism of the kinase activity and maintain higher kinase activity [33]. A recent study has revealed patients with class II mutation in colorectal cancer have worse prognosis than class-III-mutation-carrying patients. Moreover, they confirmed that patients with class I and class II BRAF showed similar poor median overall survival (OS) and disease-free survival [34]. Compared to class I and III mutations, molecular mechanism of the function of class II is less studied.

## 4. Class III BRAF Mutations

Large-scale tumor sequencing and subsequent functional analysis of individual mutations have revealed that a few mutations possess reduced BRAF kinase activity [8,35]. Class III mutations are located in the P-loop (G466), catalytic loop (N581), or DFG motif (D594, G596). One of these is the mutation at aspartic acid 594 (D594) of the DFG motif, which is a part of the activation loop [8,22,36]. In most protein kinases, the activation segment begins at the DFG motif [37], which plays a crucial role in chelating magnesium, ATP binding, and governs kinase activity [38]. BRAF D594 is located at the active site and its mutation reduces the kinase activity and has been identified in patients suffering from several types of cancer. The D594A mutation differs significantly from the V600E mutation in terms of molecular, pathological, and clinical consequences in metastatic colorectal cancer (mCRC) [18]. In melanoma, myeloma, and colorectal cancer, patients with BRAF mutations at residue D594 have a better prognosis and longer overall survival than those with the V600E mutation [18,19,20]. In mCRC, D594 mutations were exclusively associated with tumors that were microsatellite stable, unlike V600E. Co-occurrence of mutations at positions 594 and 600 of BRAF is not found, but D594 (D594N) mutation and a concomitant NRAS G13V mutation has been identified in colorectal cancer [18]. Moreover, the kinase-impaired D594 BRAF mutation is associated with the PIK3CA mutation in the mTOR pathway in melanoma [8]. Mutation in the DFG motif results in impaired kinase activity. MEK phosphorylation is completed by different MAPK/ERK protein members. BRAF activates CRAF in a RAS-independent manner. 14-3-3 binding and phosphorylation of the CRAF activation segment are required for signal transduction [39]. Studies of a murine melanoma model have revealed that tumorigenesis is closely related to the inactive BRAF mutation (D594A) and oncogenic RAS. Inhibition of the BRAF D594A mutant in the presence of oncogenic RAS results in heteromerization of BRAF/CRAF and hyperactivation of signal transduction [40].

## 5. Possible Role of BRAF-ERK-TFs in Cancer Development

Since all the BRAF pathogenic mutations, regardless of their classes, activate ERK phosphorylation, it is hypothesized that transcription factors (TFs) modulated by the ERK signaling pathway are potential downstream targets of BRAF mutations. Notably, it has been suggested that 30% of cancer tissues have constitutively activated RAS-RAF-MEK-ERK [41]. Therefore, it is important to elucidate how downstream effects of ERK phosphorylation are regulated in the context of cancer development in order to inhibit tumor development and progression. However, a limited number of studies are available to link aberrant ERK phosphorylation caused by BRAF mutation and activation of transcription factors in cancer cells. Nonetheless, several studies have identified ERK-target proteins including a number of transcription factors.

One of the best-known transcription factors that are regulated by ERK phosphorylation in cancer cells is cMyc [42]. The persistent activation of cMyc was found when it was phosphorylated at Thr58 and Ser62 [43,44]. This phosphorylation prevents protein degradation. Additionally, the mutation of cMyc at Thr58, which blocks the degradation of cMyc, has shown resistance to FGFR inhibition in several FGFR-addicted cancer cell lines [45]. These results suggest BRAF mutation-mediated ERK phosphorylation induces cMyc stability and may transfer the tumorigenic signal from BRAF mutations. Another major ERK phosphorylation-driven oncogenic transcription factor is cFos [42]. cFos is a transcription factor that forms a heterodimer with c-Jun and acts as its complex activator protein-1 (AP1). This AP1 dimer protein binds to the AP1-specific DNA sequence of promoter and enhancer regions in target genes. Besides c-Jun, cFos is known to interact with several nuclear transcription factors such as NCOA1 and SMAD3, which are regulated by ERK phosphorylation [42]. Altogether, it is assumed that BRAF mutation-mediated ERK phosphorylation can change the activity of these transcription factor-mediated gene expressions dynamically in cancer cells and exhibit tumor phenotypes.

**Figure 3:**
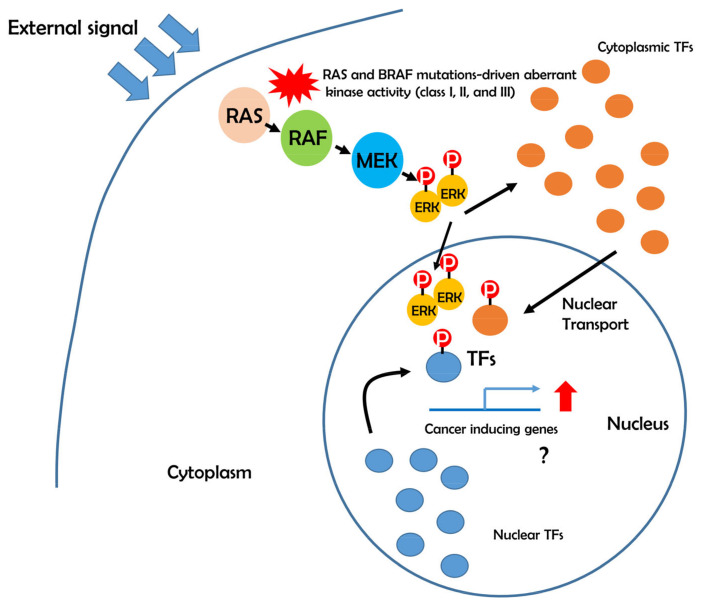
Transcription factors modulated by the ERK signaling pathway are possible downstream targets of different classes of BRAF mutations. Phosphorylated transcription factors exert their transcriptional activity in the nucleus and stimulate the cancer-inducing target gene expressions.

Apart from these well studied transcription factors, more than 100 transcription factors are determined as targets of ERK phosphorylation by an excellent analysis [42] and, among them, there are several factors that are normally localized in the outside of nucleus and transported to the nucleus after ERK phosphorylation (Figure 3). Interestingly, SMAD 1-4 are found in the list of ERK substrates that are localized in the nucleus and other organelles [42]. SMAD proteins mediate the TGF-β signaling pathway, which is known as a potent regular of epithelial to mesenchymal transition (EMT). TGF-β1 binds to its receptor II (TβRII) and activates the TGF-β receptor type I (TβRI)-kinase. This leads to phosphorylation of SMAD2 and SMAD3 in the cytoplasm [46]. Thereafter, SMAD2/3 complex is bound to SMAD4 and exerts its transactivation to express target genes. EMT is considered to be a key process in cancer metastasis in multiple cancer types [47,48]. In fact, inhibition of TGF-β signaling pathway using chemical inhibitors blocks EMT in multiple types of cancers [49,50]. These findings suggest that BRAF mutation-induced ERK phosphorylation can enhance phosphorylation of SMAD2/3 and activate EMT capacity of cancer cells.

NRF2, the protein encoded by the *NFE2L2* oncogene, is activated by ERK [51] and regulates other genes involved in oxidative stress, including heme-oxygenase and NQO1 (NRF2 major target genes; [52,53]). Several natural compounds, including Sesamin and Curcumin, have been shown to regulate oxidative stress-related genes via the ERK-NRF2 pathway [54,55]. Since the major role of BRAF is to regulate ERK-phosphorylation, NRF2 is likely regulated by BRAF and its mutant V600E form through the ERK signaling pathway in several cancers. In fact, it has been demonstrated that BRAF and RAS mutations induce *Nrf2* transcription in mouse primary cells [56]. As aberrant activation of NRF2 is found in multiple cancer types [57], further studies are needed.

During organogenesis, several transcription factors are involved as critical regulators to orchestrate cell fate decision. Interestingly, recent studies have suggested that aberrant function of these factors is highly related to tissue specific cancer development [58]. GATA transcription factors are zinc fingers containing DNA binding proteins and recognize their shared binding consensus sequence W(A/T)GATAR(A/G) [59]. They activate multiple target genes and play key roles in early development, and notably their roles in cancer development has been also reported [58]. In lung development, hepatocyte nuclear factor 3 β (HNF3β) and GATA6 transcription factors are known to be essential factors [60]. GATA6 aberrant expression is important in several cancers including lung cancer [61,62]. Moreover, GATA6 has been reported to regulate the chromatin landscape of lung cancer cells to modulate the proliferation and divergent lineage dependencies during tumor progression. HNF3β is a tumor suppressor in lung cancer, and its overexpression inhibits growth in lung cancer cells [63]. Although the role of ERK-mediated phosphorylation on HNF3β function is still unclear, the enhancement of GATA6 function through ERK phosphorylation in target gene expression is well documented in colon cancer CaCo-2 cells [64]. On the other hand, the combination of HNF4A and HNF3α, β, γ, or HNF1A/HNF3γ/GATA4 with inactivation of p19Arf can generate hepatocyte-like cells [65,66], suggesting that these factors play important roles in liver development. Liver-specific deletion of *Gata4* allele (haploinsufficiency) that inhibits GATA4 function leads to the HCC phenotype in mouse [67]. HNF4A, a known hepatic transcription factor, inhibits the ERK pathway through downregulation of phosphorylated ERK and JunD, leading to liver cirrhosis in rats [68]. HNF4A, a tumor suppressor in the liver [69], thus may act as an inhibitor of ERK-phosphorylation-mediated tumor development. Altogether, ERK phosphorylation-mediated regulation of transcription factor action is governed by a complicated regulatory network and further investigation into BRAF mutation-induced ERK phosphorylation in cancer development is required.

## 6. Aberrant Transcriptional Networks in BRAF Mutations

If BRAF-ERK-TFs pathway plays a central role in cancer development, validation of downstream BRAF target genes is required to generate more specific targets for treatment of BRAF mutation-driven tumorigenesis. In thyroid carcinoma, RNA-Seq analysis has identified that 560 genes are differentially expressed in BRAF V600E-mutated tumors compared to BRAF wild-type tumors [70]. Notably, 51 genes are downregulated and four genes (*HLAG*, *CXCL14*, *TIMP1*, and *IL1RAP*) are upregulated in immune function pathways. Thus, it is postulated that BRAF acts not only as an activator but also as a repressor. In fact, BRAF V600E has been shown to act as a regulator of the polycomb repressive complex 2 (PRC2) through upregulation of cMyc in NIH3T3 cells [71]. Thirty-three tumors from papillary thyroid cancer were immune histologically analyzed, and it was found that high levels of immunosuppressive-ligand-programmed death ligand 1 (53% vs. 12.5%) and human leukocyte antigen G (41% vs. 12.5%) were expressed in the tumors with BRAF V600E, compared to BRAF wild-type tumors [72]. Moreover, the authors found BRAF V600E tumors show both lower CD8(+) effector to FoxP3(+) regulatory T cell, and CD68(+) pan-macrophage to CD163(+) M2 macrophage, ratios, and suggested that PTC tumors with BRAF V600E displays a broadly immunosuppressive profile and disturbed host tumor immune surveillance [72]. In melanoma, BRAF V600E has been shown to regulate 1027 protein-coding transcripts; 39 annotated lncRNAs; as well as 70 unannotated, potentially novel, intergenic transcripts [73]. Moreover, they identified BRAF-regulated lncRNA1 (BANCR), and demonstrated that BANCR knockdown reduced melanoma cell migration, and the effect was rescued by the chemokine CXCL11 [73]. Other studies using colorectal cancer (CRC) cell lines and a mouse xenograft model revealed that, in the presence of BRAF V600E, the expression of growth regulation by estrogen in breast cancer protein 1 (GREB1) is highly upregulated compared to WT [74]. BRAF-mutated dysregulation of the MAPK/ERK signaling pathway results in the increased proliferation rate of CRC cells, while high expression of GREB1 may predict poor prognosis for CRC patients, suggesting GREB1 as a possible target for therapy [74]. Aberrant methylation was demonstrated in the HOXD10 promoter in papillary thyroid cancer (PTC) tissues with concomitant BRAF V600E mutation [75]. Since HOXD10 may be considered as a tumor suppressor, the hypermethylation of the promoter decreases its expression, and it may affect tumorigenesis of PTC. Furthermore, *AACS*, *ALDH3B1*, *ITPR3*, *MMD*, *LAD1*, *PVRL3*, and *RASA1* were proposed as BRAF V600E-dependent genes in thyroid carcinoma [76]. Interestingly, lower gene expression of *GADD45B* was associated with BRAF V600E, while higher GADD45B expression was connected with shorter disease-free survival in patients after total thyroidectomy and radioiodine therapy in long-term follow-up [77]. GADD45B was recognized as a marker of poor prognosis of PTC. In another study in which PTC samples harboring BRAF V600E and WT were analyzed, the upregulation of CRABP2, ECM1, and KRT17 was recognized, while MTMR3 was downregulated [78]. Since BRAF inhibitors exert non-specific effects, the identification of BRAF-specific target genes is important for the development of a cell-specific inhibitor to block BRAF mutation-mediated cancer progression. Identifying target genes and determining the level of their deregulation caused by an abnormal signaling network is critical for the successful treatment of patients with many types of cancer. The gene expression profiles of patients with BRAF V600E metastatic melanoma who showed varying progression-free survival (PFS) outcomes have been investigated [79]. Interestingly, patients with better PFS represented higher expression of immune-related genes, whereas worse PFS was found in patients with higher expression of cell cycle progression genes. In patients with shorter PFS treated with cobimetinib combined with vemurafenib, the PFS was comparable to patients with longer PFS, unlike vemurafenib-treated alone patients with shorter PFS. Furthermore, the combined therapy had no effect on upregulation of immune regulatory genes in vemurafenib-treated patients with longer PFS. These results emphasize the importance of gene profiling of individuals before applying a specific therapy. Several drugs such as vemurafenib, dabrafenib (BRAF inhibitors), and trametinib (a MEK inhibitor) have been developed to treat patients with BRAF mutations [80]. In most cases, BRAF kinase activity is enhanced by mutations, and BRAF inhibitors are expected to dampen the activity. However, the D594 mutation is known to be kinase-dead, and it enhances tumor progression by stimulating CRAF activity with oncogenic RAS [39]. Therefore, mutation type-dependent therapy should be considered to treat different classes of BRAF mutations. Up to now, a limited number of high-throughput analyses have been performed for BRAF mutations in a few cancer types. To better understand the disrupted transcriptional network caused by BRAF mutations, similar analysis should be performed in different cell types to identify cell-specific BRAF mutation-regulating genes.

## 7. Conclusions

Many studies have provided new insight into novel personalized anti-cancer therapies that target specific mutations. This highlights the importance of diverse BRAF mutations in various types of cancer. BRAF mutations in the kinase activation segment induce significant changes in the MAPK/ERK signaling pathway. The clinical impact of these mutations is locus-dependent. Within the MAPK/ERK pathway, information from external stimuli to the cell nucleus is not transduced linearly. Therefore, it is crucial to unravel the molecular mechanisms underlying signal transmission changes in the context of a specific mutation. Accordingly, molecular analysis such as RNA sequencing to identify aberrant gene expressions and transcriptional networks in the presence of different BRAF mutations should be performed in order to inform personalized anti-cancer treatment. To this end, these novel therapies should target specific mutations and their downstream effects.

## Figures and Tables

**Table 1:** BRAF D594 mutation in different type of cancers found in literature.

Mutation	Diagnosis	References
D594A	Metastatic colorectal cancer	[18]
D594E	Melanoma Multiple myeloma	[19] [20]
D594G	Metastatic colorectal cancer Non-small cell lung cancers Multiple myeloma	[18] [8] [20]
D594H	Non-small cell lung cancers	[8]
D594N	Metastatic colorectal cancer Non-small cell lung cancers Multiple myeloma	[18] [8] [20,21]

**Table 2:** Three classes of BRAF mutations identified by the International Cancer Genome Consortium (ICGC) and their clinical significance in different types of cancer.

Mutation ID	Genomic DNA Change	Location of Mutations	Clinical Significance	Donors Affected	Cancer Type
**CLASS I**					
MU62030	chr7: g.140453136 A > T	V600E	Pathogenic	814	Bladder Urothelial Cancer Chronic Lymphocytic Leukemia Colon Adenocarcinoma Colorectal Cancer Brain Glioblastoma Multiforme Head and Neck Squamous Cell Carcinoma Kidney Renal Papillary Cell Carcinoma Brain Lower Grade Glioma Liver Cancer Lung Adenocarcinoma Malignant Lymphoma Skin Cancer Pediatric Brain Cancer Rectum Adenocarcinoma Skin Adenocarcinoma Skin Cutaneous Melanoma Thyroid Cancer Head and Neck Thyroid Carcinoma
MU32987175	chr7: g.140453137 C > T	V600M	Likely pathogenic	54	Skin Cancer Skin Adenocarcinoma Skin Cutaneous Melanoma
MU40909253	chr7: g.140453136 AC > TT	V600K	Pathogenic	17	Skin Cancer
MU44780501	chr7: g.140453136 AC > CT	V600R	Pathogenic	2	Skin Cancer
MU44927644	chr7: g.140453135CA > GT	V600D	-	1	Skin Cancer
**CLASS II**					
MU1846052	chr7: g.140453134 T > C	K601E	Pathogenic	16	Chronic Lymphocytic Leukemia Lymphoid Neoplasm Diffuse Large B-Cell Lymphoma Skin Cancer Prostate Adenocarcinoma Skin Cutaneous Melanoma Gastric Adenocarcinoma Head and Neck Thyroid Carcinoma Uterine Corpus Endometrial Carcinoma
MU161538	chr7: g.140481402 C > G	G469A	Pathogenic	14	Bladder Cancer Bladder Urothelial Cancer Chronic Lymphocytic Leukemia Colon Adenocarcinoma Early Onset Prostate Cancer Lung Adenocarcinoma Lung Squamous Cell Carcinoma Oral Cancer Prostate Adenocarcinoma Skin Cutaneous Melanoma Gastric Adenocarcinoma Skin Adenocarcinoma
MU86259478	chr7: g.140481402 C > A	G469V	Pathogenic	6	Lung Adenocarcinoma Malignant Lymphoma
MU1299736	chr7: g.140481403 C > T	G469R	Likely pathogenic	6	Lung Squamous Cell Carcinoma Skin Cancer Skin Cutaneous Melanoma
MU1334968	chr7: g.140481417 C > A	G464V	Pathogenic/Likely pathogenic	3	Biliary Tract Cancer Lung Adenocarcinoma Lung Squamous Cell Carcinoma
MU4410750	chr7: g.140453145 A > T	L597Q	Pathogenic/Likely pathogenic	2	Chronic Lymphocytic Leukemia Skin Cutaneous Melanoma
MU50026	chr7: g.140453132 T > A	K601N	Likely pathogenic	2	Chronic Lymphocytic Leukemia Blood Cancer - T-Cell and Nk-Cell Lymphoma
MU44221302	chr7: g.140453146 G > C	L597V	Pathogenic	2	Biliary Tract Cancer Colon Adenocarcinoma
MU129883017	chr7: g.140481417 C > T	G464E	Pathogenic/Likely pathogenic	1	Uterine Corpus Endometrial Carcinoma
MU6236086	chr7: g.140453133 T > G	K601T	Pathogenic/Likely pathogenic	1	Gastric Adenocarcinoma
**CLASS III**					
MU126831	chr7: g.140453155 C > T	D594N	Likely pathogenic	13	Colon Adenocarcinoma Colorectal Cancer Head and Neck Squamous Cell Carcinoma Liver Cancer Lung Adenocarcinoma Lung Squamous Cell Carcinoma Malignant Lymphoma Skin Cutaneous Melanoma Gastric Adenocarcinoma
MU831694	chr7: g.140453193 T > C	N581S	Likely pathogenic	12	Biliary Tract Cancer Chronic Lymphocytic Leukemia Kidney Renal Papillary Cell Carcinoma Liver Cancer Lung Adenocarcinoma Ovarian Serous Cystadenocarcinoma Skin Cutaneous Melanoma
MU168532	chr7: g.140481411 C > A	G466V	Pathogenic/Likely pathogenic	9	Colon Adenocarcinoma Esophageal Adenocarcinoma Lung Adenocarcinoma Lung Squamous Cell Carcinoma Skin Cutaneous Melanoma
MU50763	chr7: g.140453154 T > C	D594G	Pathogenic	9	Bladder Cancer Chronic Lymphocytic Leukemia Colorectal Cancer Brain Lower Grade Glioma Malignant Lymphoma Pancreatic Cancer Pancreatic Cancer Endocrine Neoplasms
MU4440100	chr7: g.140481411 C > T	G466E	Likely pathogenic	8	Breast Er+ and Her2- Cancer Head and Neck Squamous Cell Carcinoma Skin Cancer Skin Cutaneous Melanoma
MU4420958	chr7: g.140481408 G > A	S467L	-	5	Skin Cancer Skin Cutaneous Melanoma
MU1661062	chr7: g.140481402 C > T	G469E	Pathogenic	4	Skin Cancer Oral Cancer Skin Cutaneous Melanoma
MU51987727	chr7: g.140481411 C > G	G466A	Likely pathogenic	2	Lung Adenocarcinoma Skin Cancer
MU4622596	chr7: g.140453149 C > G	G596R	Pathogenic/Likely pathogenic	2	Bladder Cancer Lung Adenocarcinoma
MU30632423	chr7: g.140453154 T > G	D594A	Likely pathogenic	2	Colorectal Cancer Liver Cancer
MU63537540	chr7: g.140453155 C > G	D594H	Pathogenic/Likely pathogenic	2	Colorectal Cancer Lung Adenocarcinoma
MU591874	chr7: g.140453148 C > T	G596D	Likely pathogenic	1	Brain Glioblastoma Multiforme
MU4622598	chr7: g.140453152 A > G	F595L	Pathogenic/Likely pathogenic	1	Bladder Cancer
